# Enhancement of Poly-γ-glutamic Acid Formation and Antioxidant Activity in *Cheonggukjang* Fermented with *Bacillus velezensis* CH7P29

**DOI:** 10.3390/antiox15080985

**Published:** 2026-08-09

**Authors:** Jong Hyoung Hong, Young Hun Jin, Shuxiang Liu, Jae-Hyung Mah

**Affiliations:** 1 Department of Food and Biotechnology, Korea University, Sejong 30019, Republic of Korea; hjh0621@korea.ac.kr (J.H.H.); younghoonjin3090@korea.ac.kr (Y.H.J.); 2 College of Food Science, Sichuan Agricultural University, Ya’an 625014, China; sliu@sicau.edu.cn

**Keywords:** fermented soybean, fermentation condition, DPPH radical scavenging activity, polyphenol, health and well-being

## Abstract

This study aimed to isolate *Bacillus* strains producing poly-γ-glutamic acid (γ-PGA), a natural antioxidant biopolymer, from *Cheonggukjang*, optimize fermentation conditions, improve γ-PGA production in the food by applying the selected strain, and investigate its potential association with antioxidant activity. Among 157 isolates, *Bacillus velezensis* CH7P29 demonstrated the highest γ-PGA production (602.80 ± 6.55 μg/mL) and was selected for fermentation. Optimal in vitro conditions for γ-PGA production were determined as 40 °C and 0–2% NaCl. In *Cheonggukjang* fermentation under optimal conditions, the CH7P29-inoculated group exhibited a marked increase in γ-PGA content (16.02 mg/g on day 4), which was significantly higher than the uninoculated control (not detected) and reference strain groups (12.25 and 11.38 mg/g, respectively). Simultaneously, its DPPH radical scavenging activity exceeded 60%, and γ-PGA content strongly correlated with antioxidant activity (R = 0.95). Conversely, total polyphenol content remained stable, indicating that γ-PGA and microbial metabolites contributed primarily to antioxidant activity. Further analysis supported this by showing that γ-PGA accounted for 37.40% of the total DPPH radical scavenging activity in the CH7P29-inoculated group at the end of fermentation. These data emphasize that *B. velezensis* CH7P29 is a promising functional starter culture for enhancing the biofunctional properties of *Cheonggukjang* and fermented soybean food.

## 1. Introduction

The global consumption of soy-based foods has continued to increase, largely because soybeans are recognized as an abundant source of health-promoting components [1]. In the late 1990s, the U.S. Food and Drug Administration (FDA) acknowledged the potential role of soy protein in reducing the risk of developing coronary heart disease by authorizing a related health claim [2]. In addition to whole soybeans, bioactive constituents derived from soy, including isoflavones and bioactive peptides, exert a wide range of beneficial biological activities, including antioxidant capacity [3], modulation of inflammatory responses [4], and inhibitory effects on cancer-related processes [5].

Beyond soybeans and nonfermented soy products, various fermented soybean foods have historically been incorporated into traditional Asian diets as vital protein sources due to their well-recognized nutritional benefits and health value [1]. For instance, *Cheonggukjang* is a rapidly fermented soybean product from Korea, produced through spontaneous or starter culture–mediated fermentation, and is typically fermented within 2–4 days [6]. The product is predominantly inhabited by various *Bacillus* species—*B. amyloliquefaciens*, *B. subtilis*, *B. coagulans*, and *B. licheniformis* [7,8,9]. Among the species, *B. velezensis* has recently emerged as a prominent and versatile starter culture in the fermentation of various foods, including traditional soybean-based products. Due to its excellent probiotic attributes, robust secretion of hydrolytic enzymes, and capacity to synthesize functional metabolites (including antimicrobial lipopeptides and poly-γ-glutamic acid), *B. velezensis* has been known to play critical roles in enhancing flavor profiles, microbial safety, and health-promoting functionalities during fermentation [10,11]. Such microbial populations are key drivers of fermentation and significantly affect the organoleptic quality and the functional properties of the resulting food. Among the various bioactive compounds in food, poly-γ-glutamic acid (γ-PGA) is a prominent functional metabolite associated with multiple health-related properties, including antioxidant capacity, glycemic regulation, and neuroprotection [12]. Structurally, γ-PGA is a high-molecular-weight polymer formed from thousands of glutamic acid isomers interlinked via γ-amide linkages. This biopolymer is valued for its excellent moisture-retention ability, antioxidant activity, and immune modulation potential [13]. In general, γ-PGA production during soybean fermentation is significantly attributed to the γ-PGA synthase activity associated with various *Bacillus* spp. [12].

In addition, fermented soybean products have been demonstrated to exhibit strong antioxidant properties [14,15]. Such activity may be attributed to γ-PGA beyond the bioactive peptides and phenolic compounds. For instance, Quach et al. [16] reported that γ-PGA possesses remarkable antioxidant capacity, as evidenced by its ability to scavenge superoxide and hydroxyl radicals and suppress lipid peroxidation, potentially through combined free-radical neutralization and metal ion–chelating mechanisms. Another study also confirmed that γ-PGA exhibits antioxidant activity against multiple radical species in vitro, thus emphasizing its potential as a natural antioxidant biopolymer [17]. Despite the recognized functional significance of γ-PGA, only limited information exists regarding the role of fermenting microorganisms—particularly γ-PGA-producing bacteria—in determining antioxidant activity in fermented soybean foods.

While current research has extensively explored the industrial production of γ-PGA and its functional properties in vitro, studies applying these findings to actual fermented food systems remain remarkably scarce [18,19]. Most existing literature has focused on the overall antioxidant capacity of fermented soy products, without independent analysis of the specific quantitative contribution of γ-PGA synthesized during fermentation [20,21]. Due to these reasons, a significant research gap exists in understanding the dynamic correlation between starter-culture-enhanced γ-PGA production and the resulting functional properties in complex food matrices like *Cheonggukjang* [22].

Therefore, this study was conducted to improve γ-PGA production during *Cheonggukjang* fermentation with selected *Bacillus* strains and determine its effects on the antioxidant activity of *Cheonggukjang*. For this purpose, *Bacillus* strains that produce high levels of γ-PGA were isolated from *Cheonggukjang*, and their capability was optimized in vitro by investigating the effects of incubation temperature and salinity. Accordingly, changes in γ-PGA levels and antioxidant activity were monitored throughout the fermentation of the selected strain-inoculated *Cheonggukjang*. Moreover, the potential contribution of γ-PGA to the overall antioxidant capacity of the product was investigated. To our knowledge, this study is the first to provide quantitative evidence that γ-PGA, whose production is improved by starter culture, may be a major contributor to the antioxidant activity in *Cheonggukjang*.

## 2. Materials and Methods

### 2.1. Bacterial Strains

A prolific γ-PGA-producing *B. velezensis* CH7P29 strain was isolated from *Cheonggukjang* in our previous study [8] and was used for improving γ-PGA production in *Cheonggukjang* in the present study. *B. subtilis*, *B. velezensis*, *B. licheniformis*, and *B. coagulans* (KCTC 3135, KCTC 13012, KCTC 1918, and KCTC 3625, respectively) type strains were obtained from the Korean Collection for Type Cultures (KCTC, Daejeon, Republic of Korea) and served as controls. *B. subtilis* KCTC 3135 and *B. velezensis* KCTC 13012 also served as controls for fermentation experiments because they demonstrated the highest and second-highest γ-PGA production capability among the type strains. Tryptic soy broth (TSB, Difco, Becton Dickinson, Sparks, MD, USA) was used for growing all the bacterial strains (incubation conditions: 37 °C for 24 h). Glycerol stocks (final concentration, 20%) of the strains were prepared using 80% glycerol solution (Junsei Chemical Co., Tokyo, Japan) and frozen at −70 °C.

### 2.2. Optimization of Conditions for In Vitro γ-PGA Production of B. velezensis CH7P29

To optimize the environmental conditions for the in vitro production of γ-PGA, *B. velezensis* CH7P29 was incubated in TSB under different conditions. After precultivation in TSB with no further addition of NaCl at 37 °C for 24 h, 100 µL of the culture was transferred to the same fresh TSB medium and incubated for 24 h at 25 °C, 30 °C, 35 °C, 37 °C, 40 °C, 45 °C, and 50 °C. To determine the effect of salinity, NaCl (≥99.0%; Samchun Pure Chemical Co., Ltd., Pyeongtaek, Republic of Korea) was added to TSB at different concentrations of 0%, 2%, 4%, 6%, 8%, and 10% (*w*/*v*), and the cultures were incubated at the selected optimal temperature (40 °C) under the same conditions. After incubation, culture broths were collected for γ-PGA analysis and total viable counts.

### 2.3. Analyses of γ-PGA Content in Bacterial Cultures and Cheonggukjang Samples

γ-PGA analysis in bacterial cultures and *Cheonggukjang* samples was conducted as described by Zeng et al. [23]. Briefly, 3 mL of bacterial cultures or 3 g of *Cheonggukjang* sample were homogenized with 15 mL of sterile deionized water using a homogenizer (T10 basic Ultra-turrax, IKA, Staufen, Germany). The homogenates were incubated at 25 °C for 1 h and centrifuged at 13,400 *g* for 20 min at 4 °C (1736R, Labogene, Seoul, Republic of Korea). After collecting the supernatant, 1.2 mL of cold absolute ethanol (≥99.9% purity, BioSesang, Seongnam, Republic of Korea) was mixed with 0.3 mL of the supernatant and incubated at 4 °C for 24 h. Then, the mixture was centrifuged at 13,400 *g* for 10 min at 4 °C and the supernatant was discarded. The pellet was dried and dissolved in 200 µL of deionized water. After centrifugation at 13,400 *g* for 20 min at 4 °C, the optical density of the supernatant was measured at 216 nm using a spectrophotometer (Lambda 35; PerkinElmer Inc., Waltham, MA, USA). The γ-PGA concentration was quantified using a calibration curve prepared with γ-PGA standard solutions at concentrations ranging from 20 to 200 μg/mL, as described by Zeng et al. [23]. Specifically, standard γ-PGA (Wako Pure Chemical Industries, Osaka, Japan) was dissolved in sterile deionized water to prepare serial standard solutions (20, 40, 60, 80, 100, 150, and 200 µg/mL), and their absorbance at 216 nm was measured in triplicate.

The method was previously validated by Zeng et al. [23], demonstrating excellent linearity (R^2^ = 0.9997), high precision (%RSD < 1.50), high recovery (>99.29%), and low limits of detection and quantitation (LOD = 0.39 μg/mL and LOQ = 1.19 μg/mL). In addition, its analytical performance was reported to be comparable to that of the HPLC method according to the International Council for Harmonisation of Technical Requirements for Pharmaceuticals for Human Use (ICH) guidelines. Therefore, the validated UV spectrophotometric method was considered appropriate for the quantitative determination of γ-PGA in the present study.

### 2.4. Cheonggukjang Fermentation with γ-PGA-Producing Bacillus Strains

Fermentation experiments of *Cheonggukjang* were performed as described by Park et al. [24]. After preparation, the soybeans were mixed with or without sterile NaCl (2%; *w*/*w*) and bacterial inocula (final concentration: ~6 log CFU/g). The inocula of *B. velezensis* CH7P29, *B. subtilis* KCTC 3135, and *B. velezensis* KCTC 13012 were prepared as described by Sinnelä et al. [25]. *Cheonggukjang* groups fermented with the type strains of *B. subtilis* KCTC 3135 or *B. velezensis* KCTC 13012 served as controls (C1 and C2, respectively), and the starter culture group fermented with *B. velezensis* CH7P29 was used as the S group. Nonfermented soybeans (B group) were also used to ensure that the fermentation experiments were performed aseptically. The fermentation process was conducted at 40 °C over a period of 4 days. During fermentation, 20 g from each soybean group was collected every 24 h, and several basic fermentation parameters (see Section 2.5), γ-PGA content, and antioxidant capacity (as detailed in Section 2.6) were determined.

### 2.5. Analysis of Basic Fermentation Parameters

To ensure that the *Cheonggukjang* groups were fermented properly, basic fermentation parameters were measured as described previously [8]. Briefly, the pH and a_w_ of the samples were measured using an Orion 3-star pH Benchtop meter (Thermo Scientific, Waltham, MA, USA) and an AquaLab Pre hygrometer (Meter Group, Inc., Pullman, WA, USA), respectively. Salt content was determined using Official Method 960.29 [26]. Total mesophilic viable bacterial counts of bacterial cultures and *Cheonggukjang* groups were determined using plate count agar (Difco).

### 2.6. Analysis of Texture Profiles

Texture profile analysis was performed with slight modifications based on the method described by Zeng et al. [23]. Fifteen *Cheonggukjang* soybean kernels with similar size were randomly selected for each measurement. The samples were subjected to a two-cycle compression test using a texture analyzer (Zwick/Roell, Ulm, Germany) equipped with a cylindrical compression probe. The compression strain was set at 30% with a test speed of 100 mm/min, and the probe returned to the initial position between cycles. All measurements were performed in triplicate.

### 2.7. Analyses of Antioxidant Activities of Cheonggukjang

#### 2.7.1. Analysis of Total Polyphenol Content

The total polyphenol content of *Cheonggukjang* groups was determined following the method developed by Zhu et al. [27] and slightly modified by Jin et al. [8]. Briefly, 5 g of the *Cheonggukjang* products were homogenized with 45 mL of deionized water and filtered through a 0.2 μm pore-size syringe filter (Millipore Co., Bedford, MA, USA). Then, 200 μL of the homogenate or standards were mixed with 750 μL of Folin and Ciocalteu’s phenol reagent (50%; Sigma Chemical Co., St. Louis, MO, USA) and incubated at room temperature for 3 min. Then, the solution was mixed with 600 μL of sodium carbonate (2%; Junsei Chemical Co., Tokyo, Japan) reagent and incubated at room temperature for 30 min in the dark. After that, the absorbance of 1 mL of the solution was measured at 750 nm using a Lambda 35 spectrophotometer (PerkinElmer Ltd., Waltham, MA, USA). Gallic acid (GA; Sigma) solutions with concentrations of 40, 80, 120, 160, and 200 μg/mL were used to generate the calibration curve (R^2^ > 0.99).

#### 2.7.2. Analysis of 1,1-Diphenyl-2-picrylhydrazyl (DPPH) Radical Scavenging Activity

The DPPH radical scavenging activity of *Cheonggukjang* groups was evaluated using the DPPH radical scavenging capacity assay, as described by Jeng et al. [28] and slightly modified by Jin et al. [8], which has been widely proposed as a representative method for analyzing the antioxidant activity of plant extracts [29,30]. In detail, a 1 mM DPPH radical solution was prepared by dissolving DPPH (Sigma) in 4 mL of methanol (SK chemicals, Ulsan, Republic of Korea). Then, 25 μL of the homogenate (prepared in Section 2.7.1) was mixed with 0.5 mL of the solution. After incubation at room temperature for 30 min, the absorbance of 200 μL of the mixture was measured at 517 nm with a microplate reader. Ascorbic acid (Sigma) was used as a positive control. The DPPH radical scavenging activity (%) was expressed as [1 − sample absorbance/control absorbance] × 100. In this study, the antioxidant activity was assessed solely by the DPPH radical scavenging assay, as it is commonly used as a representative approach to determine antioxidant capacity in plant extracts [29,30].

A standard curve was used to estimate the DPPH radical scavenging activity of γ-PGA in all *Cheonggukjang* groups. The standard curve was generated using the filtrates prepared from the working standard solutions of γ-PGA at concentrations of 0–20 mg/mL. The γ-PGA content in the *Cheonggukjang* groups was converted into DPPH radical scavenging activity using the standard curve plotting the concentration of γ-PGA versus the DPPH radical scavenging activity of γ-PGA. The DPPH radical scavenging activity of other substances was derived by subtracting that of γ-PGA from the total activity of *Cheonggukjang* groups.

### 2.8. Statistical Analysis

Data were obtained from triplicate experiments and are reported as mean values with corresponding standard deviations. Statistical significance was analyzed in Minitab (version 17; Minitab Inc., State College, PA, USA) using one-way ANOVA, and Fisher’s pairwise comparisons were applied. Significant differences were indicated using a threshold of *p* < 0.05. A linear correlation analysis between the DPPH radical scavenging activity and γ-PGA content of the inoculated *Cheonggukjang* groups was also performed using the same statistical software.

## 3. Results and Discussion

### 3.1. γ-PGA Production of Bacillus Strains Originated from Cheonggukjang

To elucidate the microbial factors responsible for γ-PGA production during *Cheonggukjang* fermentation and to screen the starter culture for enhancing γ-PGA formation in the food, this study focused on γ-PGA-producing *Bacillus* strains. This approach was adopted because (i) *Bacillus* spp. are widely recognized as key microorganisms in the fermentation of soybean-derived foods [7,8,9] and (ii) most *Bacillus* spp. have been reported to have γ-PGA production capability [12]. Based on this rationale, 157 *Bacillus* strains isolated from *Cheonggukjang* were individually evaluated for their γ-PGA-producing capacity. Type strains of various *Bacillus* strains, including *B. velezensis* KCTC 13012, *B. subtilis* KCTC 3135, *B. coagulans* KCTC 3625, and *B. licheniformis* KCTC 1918, served as positive controls.

**Figure 1:**
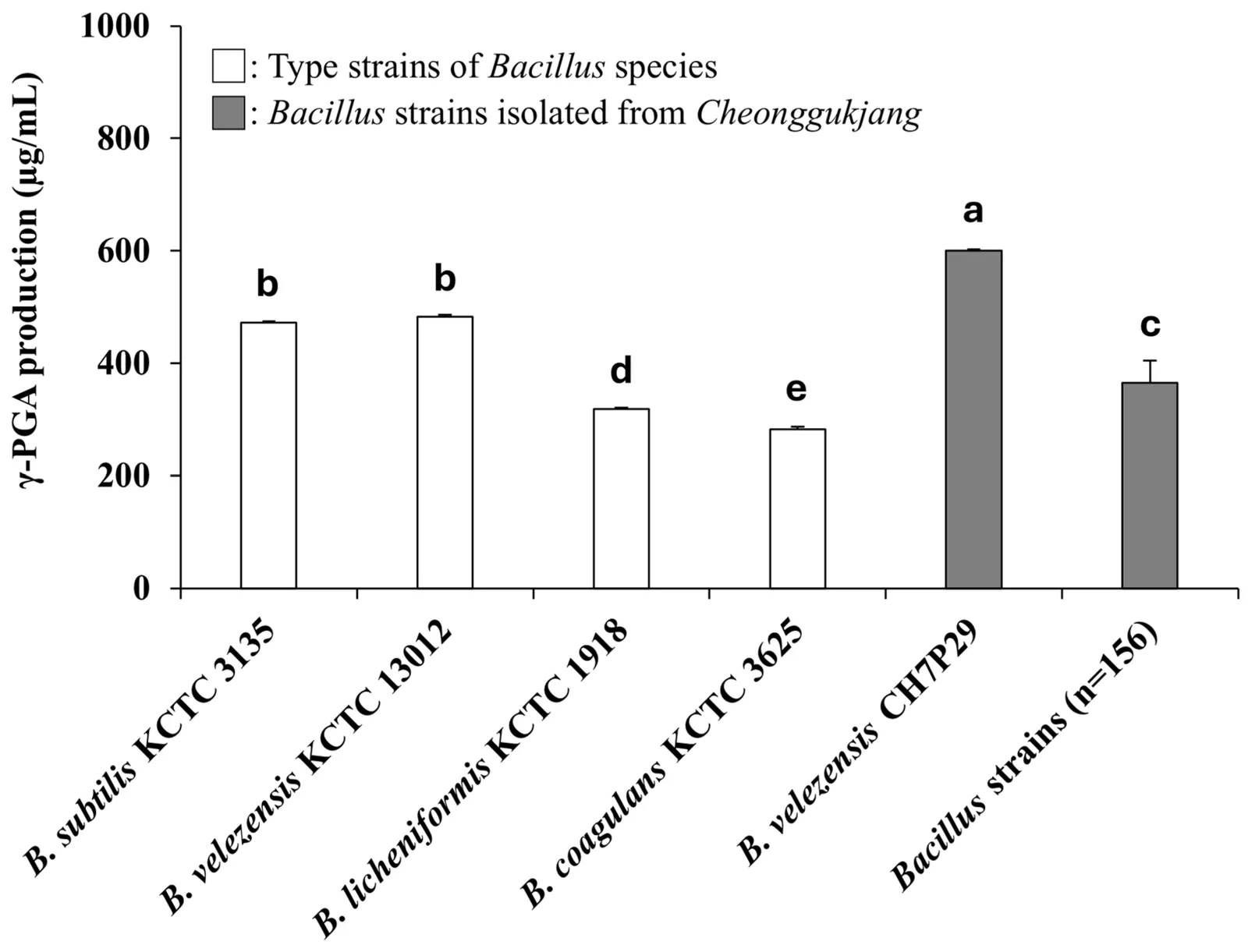
γ-PGA production by various *Bacillus* strains in assay media. Values of bars with different letters (a–e) are significantly different (*p* < 0.05) according to Fisher’s pairwise comparisons. The number (n) indicates *Bacillus* strains isolated from *Cheonggukjang* products. Error bars indicate standard deviations determined from triplicate experiments.

As shown in Figure 1, strain CH7P29, confirmed as *B. velezensis* by 16S rRNA analysis, produced the greatest amount of γ-PGA (602.80 ± 6.55 μg/mL, mean ± standard deviation) compared with both reference strains and other isolates. Of the positive controls, *B. subtilis* KCTC 3135 and *B. velezensis* KCTC 13012 produced the highest level of γ-PGA (524.33 ± 2.69 and 527.11 ± 4.56 μg/mL, respectively), followed by *B. licheniformis* KCTC 1918 (332.45 ± 4.02 μg/mL) and *B. coagulans* KCTC 3625 (302.77 ± 8.22 μg/mL). The other *Bacillus* isolates exhibited γ-PGA production (368.48 ± 30.05 μg/mL, mean ± standard deviation of γ-PGA production of multiple strains). Therefore, *B. velezensis* CH7P29 was selected for further in situ fermentation trials.

The *Bacillus* species used in this study—*B. velezensis*, *B. subtilis*, *B. coagulans*, and *B. licheniformis*—have been reported to be autochthonous in soybean-based products and have been widely applied to enhance or optimize fermented soybean foods [9,31]. Such species have also been considered prolific γ-PGA producers [12]. In particular, Liu et al. [32] reported that a *B. velezensis* strain isolated from Chinese traditional fermented soybean food, *Douchi*, produced high levels of γ-PGA. Consistent with these findings, *B. velezensis* CH7P29 exhibited significantly greater γ-PGA production in assay media than the tested reference strains belonging to *B. velezensis*, *B. subtilis*, *B. coagulans*, and *B. licheniformis*. Furthermore, despite being isolated from the same source (*Cheonggukjang*), the *Bacillus* strains exhibited considerable variability in γ-PGA production, with a standard deviation of 30.05 μg/mL. Taken together with previous reports, these results suggest that γ-PGA productivity is affected by not only species differences but also strain-specific characteristics [33,34].

Consequently, these findings indicate that γ-PGA-producing *Bacillus* strains isolated from soybean-based foods such as *Cheonggukjang* could be effectively utilized to enhance γ-PGA accumulation in fermented soybean products. Moreover, γ-PGA-enriched fermented soybeans may represent a promising resource for the development of natural antioxidant ingredients with potential cosmeceutical and nutraceutical applications.

### 3.2. Optimization of γ-PGA Production Conditions of B. velezensis CH7P29 in Bacterial Culture

According to previous studies on γ-PGA production by some *Bacillus* strains [28,29,30], their γ-PGA production was significantly affected by temperature and salinity. Therefore, in the present study, the effects of incubation temperature (25–50 °C) and NaCl concentration (0–10%) on the γ-PGA production of the selected *B*. *velezensis* CH7P29 were tested in the assay media.

As shown in Table 1, γ-PGA production significantly increased from 478.64 to 596.48 µg/mL (*p* < 0.05) when the incubation temperature increased from 25 to 40 °C. Above this temperature (i.e., 45 and 50 °C), the production dramatically decreased to 431.18 and 415.44 µg/mL, respectively. Conversely, total viable counts steadily decreased from about 8.2 log CFU/mL to 7.2 log CFU/mL as incubation temperature increased. This result suggests that the growth of *B*. *velezensis* CH7P29 may be less related to γ-PGA production. Similar trends have been reported in a previous study by Zeng et al. [35] where a *B. subtilis* strain produced more γ-PGA but exhibited less growth when the incubation temperature increased from 28 to 50 °C. Although the dynamic nature of the relationship between the growth and γ-PGA production of *Bacillus* spp. remains unknown, in this study, 40 °C was selected as the optimal incubation temperature.

Regarding the effect of salinity, γ-PGA production was highest with statistical significance at 0% and 2% NaCl concentrations (562.44 ± 2.36 and 570.22 ± 4.36 µg/mL, respectively), whereas it gradually decreased to 23.11 µg/mL as the NaCl concentration increased to 8% (Table 2). At 10% NaCl concentration, the production was not detected. Meanwhile, total viable counts were generally stable (approximately 8 log CFU/mL) irrespective of NaCl concentration, suggesting that *B. velezensis* CH7P29 may be halotolerant. Several studies have also reported that certain strains of *B. velezensis* exhibit halotolerance, maintaining survival and activity under elevated salt concentrations [36,37].

In the present study, γ-PGA production by *Bacillus* spp. decreased when salinity increased from 2 to 10%. Although this increase in salinity is generally expected to inhibit both microbial growth and γ-PGA production [38], no growth inhibition was found in the current study. In contrast, some previous studies have reported that high NaCl concentrations stimulated γ-PGA production by *Bacillus* strains. For instance, Wei et al. [39] observed that *B. licheniformis* WX-02 produced the highest γ-PGA content at 8% NaCl, whereas its growth was lowest under the same condition. In a further study from the same research group, they showed that osmotic stress induced the expression of the γ-PGA synthetase gene in the same strain [40]. Nevertheless, supporting our results, another previous study found that some *Bacillus* spp., *B. sonorensis*, *B. subtilis*, and *B. licheniformis*, originating from various fermented soybean foods, exhibited reduced γ-PGA production with decreased bacterial growth as salinity increased from 0% to 8% [41]. Based on the previous and current studies, the effect of salinity on γ-PGA production by *Bacillus* strains may be strain- and species-dependent. Although the dynamic relationship between bacterial growth and γ-PGA production in *Bacillus* spp. remains unclear, 0% and 2% concentrations of NaCl were selected as the optimal salinity conditions in the present study. Consequently, the optimal conditions to enhance γ-PGA production by *B. velezensis* CH7P29 in bacterial culture were determined to be 40 °C with 0% or 2% NaCl.

### 3.3. γ-PGA Production by B. velezensis CH7P29 in Cheonggukjang Fermented Under Optimized Conditions

To examine the practical effects of *B. velezensis* CH7P29 and optimal conditions (40 °C; 0% or 2% NaCl) on γ-PGA formation, *Cheonggukjang* fermentation experiments were conducted. *B. subtilis* KCTC 3135 or *B. velezensis* KCTC 13012-inoculated *Cheonggukjang* groups served as controls (C1 and C2, respectively) based on the following reasons: (i) because *B. subtilis* is generally considered fermenting bacterium in *Cheonggukjang* [31], *B. subtilis* type strain was used as an inoculum for the first control (C1 group), and (ii) because *B. velezensis* has also been reported to be indigenous *Bacillus* spp. in food and other fermented soybean foods [42,43], *B. velezensis* type strain-inoculated group (C2 group) was used as a counterpart bacterium of the selected strain-inoculated group (S group) for the second control. Non-fermented soybean (B group) was also used to ensure that the fermentation experiments were performed aseptically. To demonstrate that the fermentation of *Cheonggukjang* progressed properly, basic fermentation parameters (pH, a_w_, and total viable mesophilic bacterial counts) and texture profiles, as well as γ-PGA content in all groups, were determined every day.

**Figure 2:**
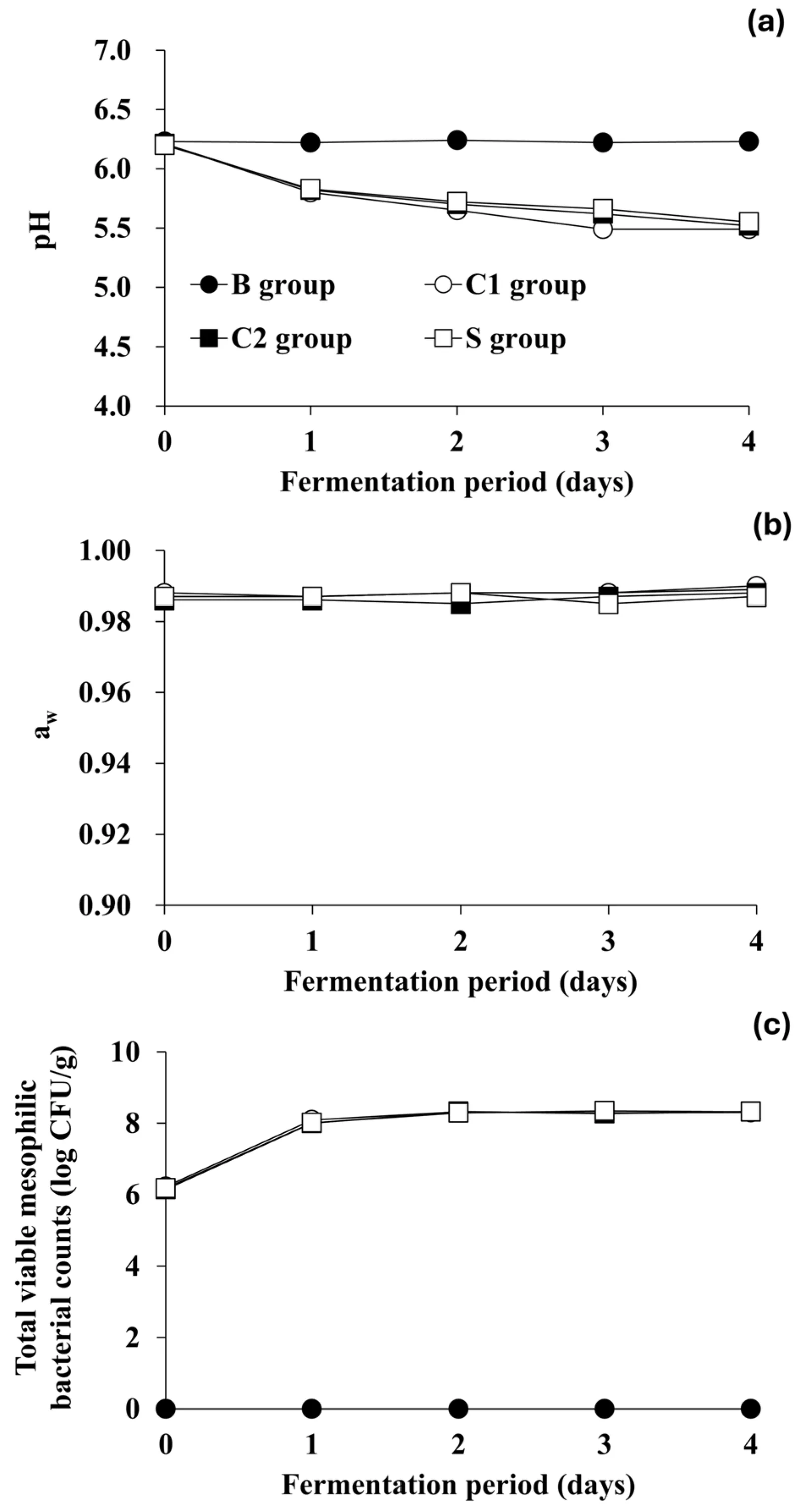
Changes in (**a**) pH, (**b**) water activity, and (**c**) total viable mesophilic bacterial counts during fermentation of *Cheonggukjang* inoculated with γ-PGA-producing *Bacillus* strains under optimal conditions of 40 °C and 2% NaCl. ●: B group prepared without an inoculum (blank), ○: C1 group inoculated with *B. subtilis* KCTC 3135 (control 1), ■: C2 group inoculated with *B. velezensis* KCTC 13012 (control 2), □: S group inoculated with *B. velezensis* CH7P29. Error bars indicate standard deviations determined from triplicate experiments.

As a result of analyzing the fermentation parameters, texture parameters and γ-PGA content in *Cheonggukjang*, the parameters in *Cheonggukjang* prepared with 0% and 2% NaCl concentrations were similar, and the γ-PGA content was higher at 2% NaCl than at 0% NaCl (Figure S1). Hence, based on these observations, the fermentation results at 40 °C with the addition of 0% NaCl are not described hereafter. As shown in Figure 2a, the pH of all groups was 6.22–6.28 at day 0. Although the pH of the B group remained constant until the end of fermentation, that of the other groups decreased to 5.56–5.75. Regarding a_w_, the values in all groups were 0.980–0.984 at day 0; their levels stayed constant (Figure 2b). In Figure 2c, total mesophilic viable bacterial counts in the inoculated groups were about 6 log CFU/g at day 0 and increased to about 8 log CFU/g on day 1. The counts in the inoculated groups remained constant thereafter. As for the texture profile, as shown in Figure S2, all measured parameters (hardness, adhesiveness, springiness, gumminess, chewiness, and cohesiveness) of all inoculated groups were very similar to each other throughout fermentation, while the B group remained almost unchanged (except for hardness, which gradually decreased but remained at a slightly higher level than all inoculated groups). In addition, after fermentation, all inoculated groups had parameters similar to those of typical commercial *Cheonggukjang* products (Table S1). Based on the basic fermentation parameters and texture parameters, it was determined that all the inoculated groups fermented properly.

**Figure 3:**
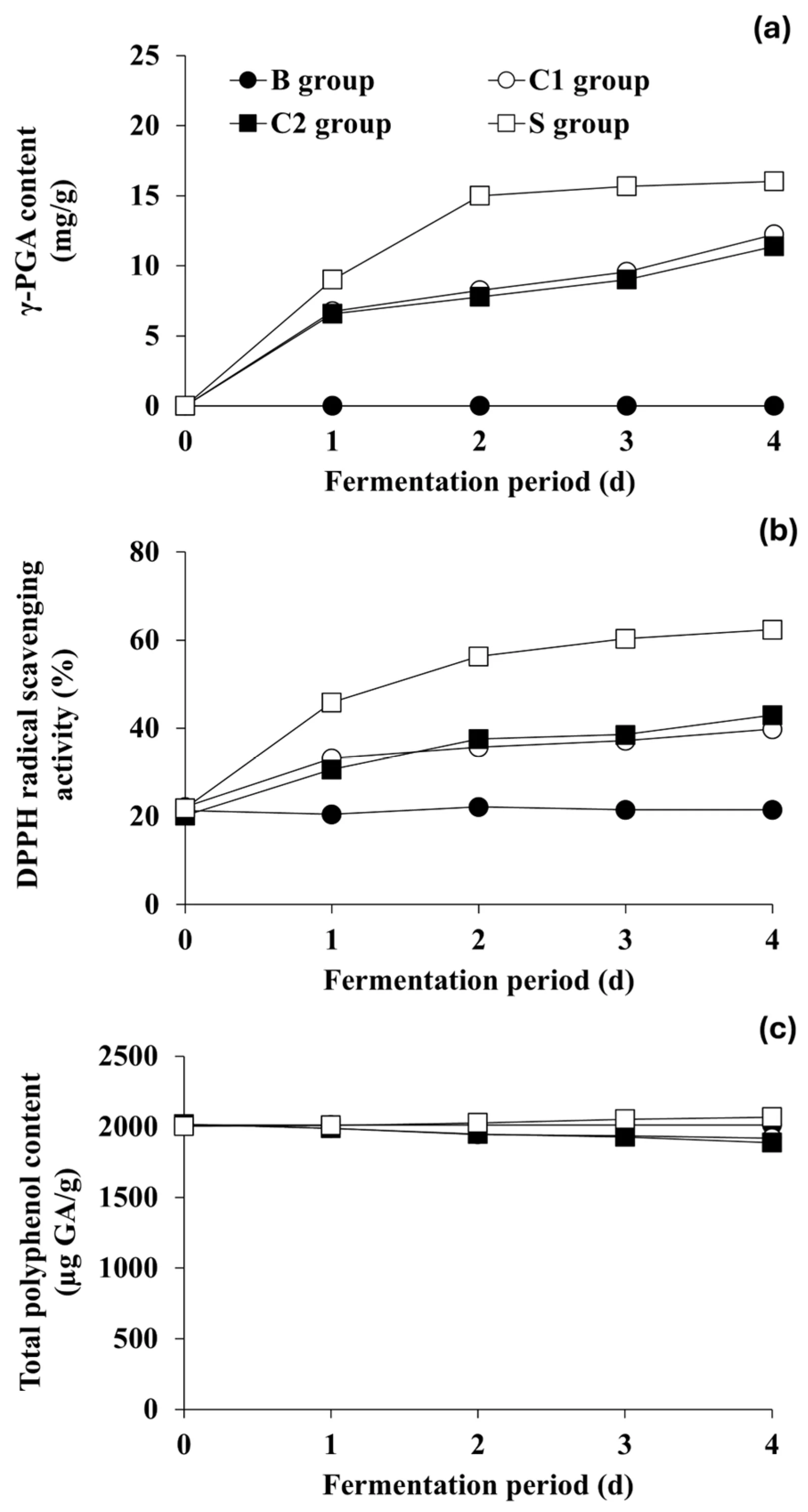
Changes in (**a**) γ-PGA content, (**b**) DPPH radical scavenging activity, and (**c**) total polyphenol content during fermentation of *Cheonggukjang* inoculated with γ-PGA-producing *Bacillus* strains under optimal conditions. ●: B group prepared without an inoculum (blank), ○: C1 group inoculated with *B. subtilis* KCTC 3135 (control 1), ■: C2 group inoculated with *B. velezensis* KCTC 13012 (control 2), □: S group inoculated with *B. velezensis* CH7P29. Error bars indicate standard deviations determined from triplicate experiments.

In Figure 3a, the γ-PGA content in all the inoculated *Cheonggukjang* groups (C1, C2, and S groups) gradually increased as fermentation progressed, while that in the non-inoculated group (B group) was not detected. The γ-PGA content in the S group was highest (16.02 mg/g) by day 4, whereas that in the C1 and C2 groups was 12.25 and 11.38 mg/g, respectively. These results indicate that γ-PGA formation depends on microbial activity. Furthermore, the γ-PGA content in the S group after fermentation was also much higher than the average value (11.96 mg/g) reported for commercial *Cheonggukjang* products manufactured in different provinces in South Korea [8]. A previous study found that starter-inoculated *Cheonggukjang* had a significantly higher γ-PGA content than naturally fermented *Cheonggukjang* [44], which, together with results of the current study, highlights that starter application can increase γ-PGA content in *Cheonggukjang*. Moreover, the S group exhibited the highest γ-PGA accumulation at the end of fermentation, which was substantially higher than that observed in the C1 and C2 groups. These results clearly demonstrate that γ-PGA formation during *Cheonggukjang* fermentation is strongly strain-dependent, consistent with previous studies reporting significant variability in γ-PGA productivity among *Bacillus* strains isolated from fermented soybean foods [12]. Moreover, the sustained increase in γ-PGA levels over the fermentation period further suggests that the selected fermentation conditions were favorable for γ-PGA biosynthesis, consistent with previous reports showing that the ability of *Bacillus* spp. to produce γ-PGA was largely influenced by strain characteristics and environmental factors such as temperature and salinity [33,34].

Based on these findings, the use of the selected γ-PGA-producing *Bacillus* strain (i.e., *B. velezensis* CH7P29), together with optimized fermentation conditions, may enhance the functional properties of *Cheonggukjang* and other fermented soybean foods.

### 3.4. Changes in Antioxidant Activity During Cheonggukjang Fermentation with γ-PGA Production by B. velezensis CH7P29

To investigate the contribution of γ-PGA content to antioxidant activity, the relationships between γ-PGA production, total polyphenol content, and DPPH radical scavenging activity were analyzed during *Cheonggukjang* fermentation. As mentioned in a previous section, γ-PGA formation significantly increased over the 4-day fermentation period, reaching a maximum of approximately 16 mg/g in the S group, compared to negligible levels in the B group (uninoculated control). The C1 and C2 groups also showed moderate γ-PGA accumulation (about 12 mg/g), indicating that *Bacillus* strains significantly influenced γ-PGA production.

**Figure 4:**
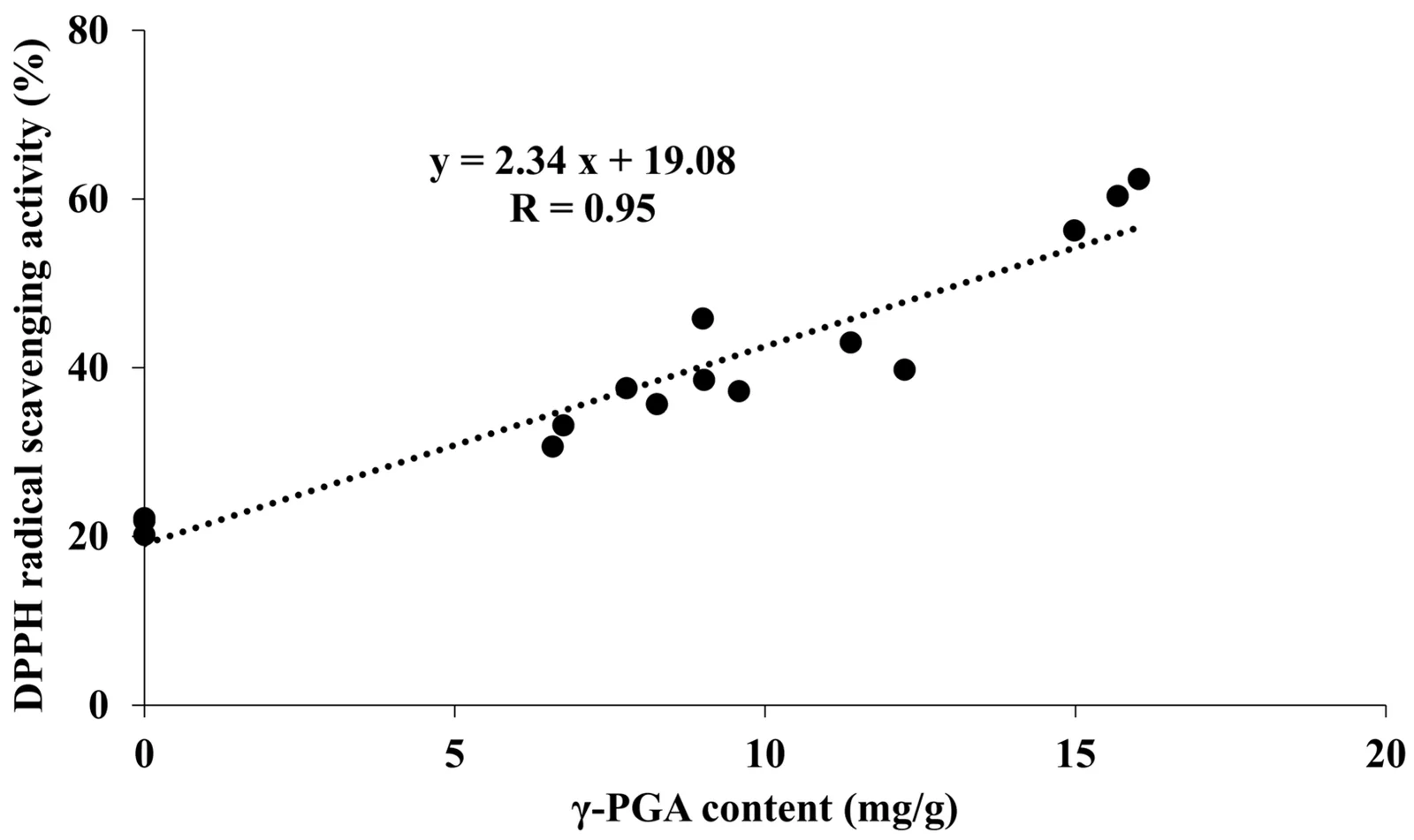
The linear regression between γ-PGA content and DPPH radical scavenging activity in *Cheonggukjang* inoculated with γ-PGA-producing *Bacillus* strains under optimal conditions. Each data point indicates the average obtained in three independent experiments.

In Figure 3b, the initial DPPH radical scavenging activity in all groups was about 20%. Except for that in the B group, the scavenging activity in all the inoculated groups increased throughout the fermentation period. Particularly, the S group had the highest DPPH radical scavenging activity over 60% by day 4. This was significantly higher than those in the other groups, which remained below 40%. This increase in antioxidant capacity is consistent with the elevated γ-PGA production. Supporting this speculation, a linear regression analysis showed a very strongly significant positive correlation (R = 0.95) between γ-PGA content and DPPH radical scavenging activity (Figure 4). In contrast, the total polyphenol content remained relatively stable across all groups throughout fermentation, with minor fluctuations around 2000 µg GA/g (Figure 3c). This suggests that the changes in antioxidant activity were not due to polyphenol fluctuations, but rather associated with γ-PGA and probably other microbial metabolites. In addition, the remarkable stability of the total polyphenol content observed in this study is a distinctive result when compared to other fermented food systems, such as fermented dairy or certain vegetable fermentations. While certain lactic acid bacteria (LAB) or fungi in those systems can extensively alter, metabolize, or degrade polyphenolic structures during fermentation [45], *Bacillus* spp. typically involved in soybean fermentation tend to preserve the overall phenolic framework without aggressive degradation [46]. Furthermore, moderate temperature and low-salinity conditions applied to maximize γ-PGA biosynthesis may minimize the oxidative or thermal degradation of native soybean polyphenols [47,48].

**Figure 5:**
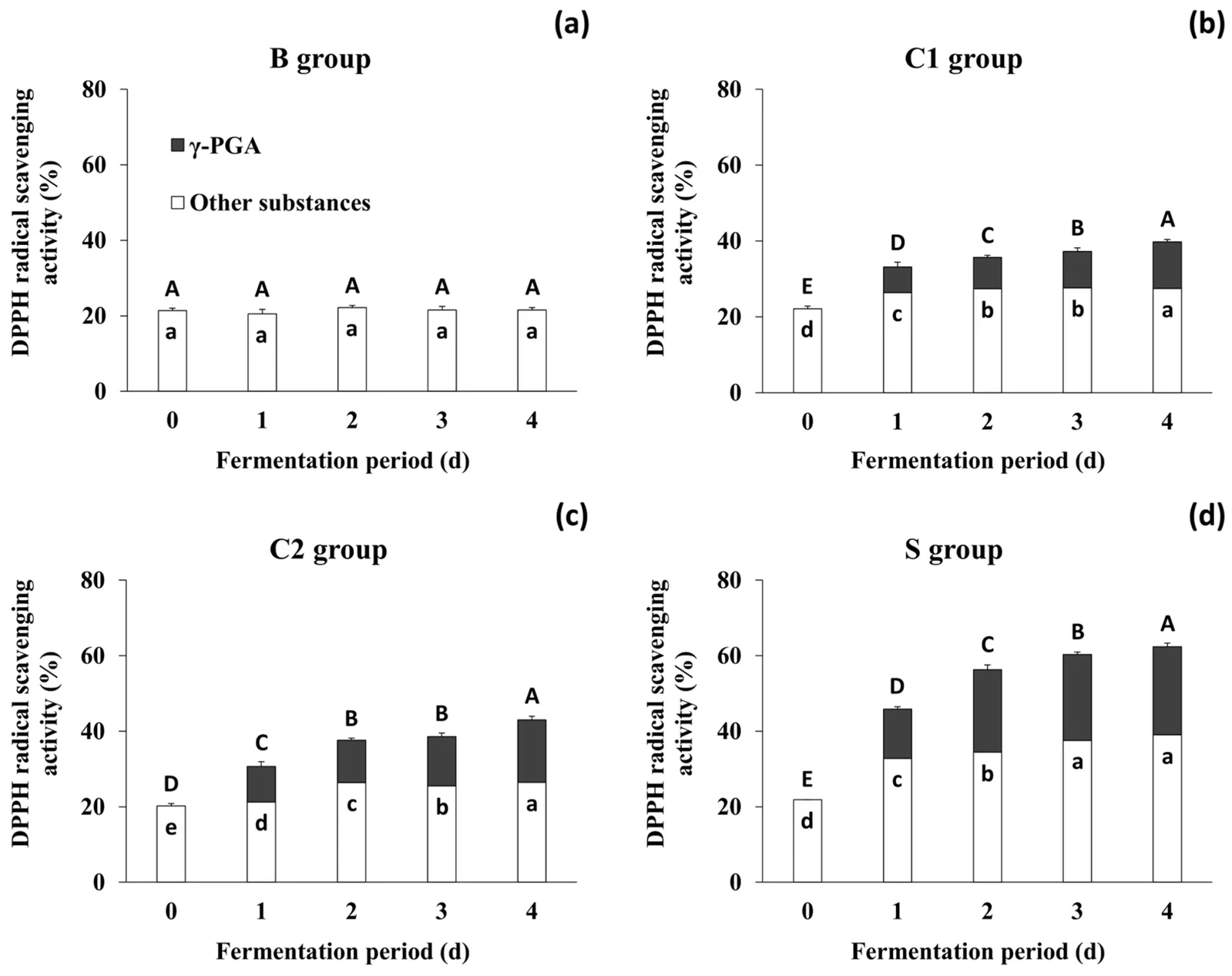
Changes in DPPH radical scavenging activity during fermentation of *Cheonggukjang* inoculated with γ-PGA-producing *Bacillus* strains under optimal conditions. (**a**) B group prepared without an inoculum (blank), (**b**) C1 group inoculated with *B. subtilis* KCTC 3135 (control 1), (**c**) C2 group inoculated with *B*. *velezensis* KCTC 13012 (control 2), (**d**) S group inoculated with *B*. *velezensis* CH7P29. γ-PGA content in *Cheonggukjang* groups was converted to DPPH radical scavenging activity by referring to a standard curve, and the activity of other substances was derived by the subtraction of that of γ-PGA from the total activity of *Cheonggukjang* groups. ⬒: total DPPH radical scavenging activity of *Cheonggukjang* groups, ■: DPPH radical scavenging activity of γ-PGA in *Cheonggukjang* groups, □: DPPH radical scavenging activity of other substances in *Cheonggukjang* groups. The mean values of the total DPPH radical scavenging activity (full bars, capital letters, A–E) or the activity of γ-PGA (black bars, small letters, a–e) that are not followed by the same letter are significantly different (*p* < 0.05) according to Fisher’s pairwise comparisons. Error bars indicate standard deviations determined from triplicate experiments.

To clarify the relative contribution of γ-PGA to the antioxidant activity of *Cheonggukjang*, the total DPPH radical scavenging activity was divided into the contribution of γ-PGA and that of other metabolites (Figure 5). As described right above, the B group exhibited the lowest total DPPH radical scavenging activity throughout the fermentation, with no contribution from γ-PGA. Conversely, the S group reached the highest total DPPH radical scavenging activity on the 4th day of fermentation, with the contribution of γ-PGA content accounting for 37.40% of the activity. The other inoculated groups (C1 and C2 groups) showed moderate activity, with the contribution of γ-PGA accounting for 30.81 and 38.42%, respectively, on the same day. These observations showed that the increase in the total DPPH radical scavenging activity during fermentation was largely attributable to γ-PGA formation, particularly in the S group. Conversely, the contribution of other substances remained relatively constant or showed minor increases across all groups, indicating that other substances played a secondary role in the overall antioxidant capacity. This trend was consistent with the relatively stable total polyphenol content observed during fermentation (Figure 3c). As such, the significant contribution of γ-PGA to the overall antioxidant capacity compared to other substances such as polyphenols can be elucidated through its specific biochemical mechanisms. Structurally, the abundant carboxyl groups arrayed along the γ-PGA polymer chain provide a highly anionic environment capable of interacting with various pro-oxidant factors, thereby effectively suppressing oxidative chain reactions [13]. In addition, the high density of negative charges derived from the carboxylate groups endows γ-PGA with a robust metal-chelating capacity. By sequestering transition metal ions (such as Fe^2+^ and Cu^2+^), γ-PGA effectively inhibits the generation of reactive oxygen species via the Fenton reaction, thereby exerting profound indirect antioxidant effects [49].

γ-PGA plays a crucial role in improving the texture and viscosity of fermented soybean products and is known for its water-holding and emulsifying properties [16,29]. In the present study, the substantial increase in γ-PGA level observed in the S group indicates the strong metabolic capacity of the selected *B. velezensis* CH7P29 strain to produce γ-PGA during *Cheonggukjang* fermentation. Therefore, the significantly higher γ-PGA level in the S group (30.78% and 40.77%, respectively) than in the C1 and C2 groups clearly demonstrates that *B. velezensis* CH7P29 is a promising starter candidate suitable for fermented soybean foods. Beyond these basic physicochemical functions, γ-PGA has recently attracted increasing attention for its potential health-promoting effects, including antioxidant activity [12] that is beneficial for protective and therapeutic implications in obesity [50,51], diabetes [52], various types of cancers such as ovarian and breast cancers [53], and neurodegenerative disorders such as Alzheimer’s disease and other forms of cognitive decline [12].

Altogether, this study provides direct evidence that γ-PGA may be one of the major functional contributors to antioxidant activity in *Cheonggukjang*, which is supported by a strong correlation observed between γ-PGA content and DPPH radical scavenging activity. Therefore, the fermentation of *Cheonggukjang* by the γ-PGA-producing *B*. *velezensis* CH7P29 under optimal conditions may contribute to reinforcing functional potential (such as anticancer, anti-obesity, and anti-neurodegenerative activity) by enriching *Cheonggukjang* with γ-PGA.

The DPPH assay is highly useful for high-throughput screening at room temperature due to its simple preparation and excellent radical stability [54]. However, compared to ABTS and FRAP assays, it exhibits several mechanistic limitations: steric hindrance from its bulky molecular structure [55,56], low reactivity with hydrophilic components due to organic solvent dependence [57], strict neutral pH constraints, and an inability to measure iron-reducing mechanisms [45]. Despite some of these limitations, previous studies [29,30] suggested that the DPPH radical scavenging assay is suitable for evaluating the antioxidant activity of plant extracts. These studies also found a strong positive correlation between methods such as DPPH, FRAP, and ABTS used to analyze antioxidant activity, suggesting that evaluating activity using multiple assays may be superfluous. However, for industrial applications, antioxidant activity should generally be evaluated using multiple assays and further validated through in vivo and clinical trials.

## 4. Conclusions

In this study, *B. velezensis* CH7P29 was confirmed as a highly prolific γ-PGA-producing strain compared to both reference strains and other *Bacillus* strains from *Cheonggukjang*. Further optimization of culture conditions revealed that γ-PGA production by *B. velezensis* CH7P29 was highest at 40 °C and under low-salinity conditions (0–2% NaCl). When applied to in situ fermentation, γ-PGA content in the *B. velezensis* CH7P29-inoculated group was much higher than that in the *B. subtilis* and *B. velezensis* type strain-inoculated groups (C1 and C2 groups; 30.78% and 40.77%, respectively). The fermentation results also showed that the *B. velezensis* CH7P29-inoculated group exhibited significantly higher antioxidant activity (56.84% and 45.09%, respectively) than the C1 and C2 groups. Further analysis revealed that γ-PGA accounted for a substantial proportion of the total antioxidant activity, whereas other substances played less important roles in the antioxidant activity.

Overall, this study underscores the importance of strain selection and fermentation condition optimization in enhancing the functional properties of traditional fermented soybean foods. The use of *B. velezensis* CH7P29 could result in the development of *Cheonggukjang* with enriched γ-PGA content and potentially associated health-promoting benefits (such as anticancer, anti-obesity, and anti-neurodegenerative activity) without altering the polyphenol levels. Consequently, this approach offers a promising strategy for the advancement of functional fermented foods and supports the potential application of γ-PGA-producing *Bacillus* strains in the functional food industry.

Despite these promising results, this study has certain limitations. The antioxidant capacities were primarily evaluated using chemical-based in vitro assays, and a comprehensive sensory evaluation is required to fully determine consumer acceptability. Therefore, future perspectives should include in vivo physiological validations and extensive organoleptic profiling.

## Figures and Tables

**Table 1:** Effects of incubation temperatures on γ-PGA production of *B. velezensis* CH7P29 strain.

Incubation Temperatures (°C)	Total Viable Counts (Log CFU/mL)	γ-PGA Production (µg/mL)
25	8.25 ± 0.02 ^A,1^	478.64 ± 7.56 ^E,2^
30	7.97 ± 0.12 ^B^	523.56 ± 5.88 ^D^
35	7.39 ± 0.12 ^C^	554.11 ± 2.59 ^C^
37	7.97 ± 0.09 ^B^	587.56 ± 3.15 ^B^
40	7.33 ± 0.04 ^C^	596.48 ± 6.16 ^A^
45	7.45 ± 0.12 ^C^	431.18 ± 2.33 ^F^
50	7.24 ± 0.02 ^C^	415.44 ± 4.50 ^G^

^1^ Data represent mean ± standard deviation determined by triplicate experiments. Mean values with different upper letters (A–G) in the same column are significantly different (*p* < 0.05) according to Fisher’s pairwise comparisons. ^2^ The correlation between incubation temperature (X, °C) and γ-PGA production (Y, μg/mL) was described by the quadratic equation Y = −0.84X^2^ + 60.02X − 501.00 (R = 0.88).

**Table 2:** Effects of NaCl concentrations on γ-PGA production of *B. velezensis* CH7P29 strain.

NaCl Concentrations (%)	Total Viable Counts (Log CFU/mL)	γ-PGA Production (µg/mL)
0	7.88 ± 0.17 ^A,1^	562.44 ± 2.36 ^A,2^
2	7.99 ± 0.02 ^A^	570.22 ± 4.36 ^A^
4	7.43 ± 0.15 ^B^	555.15 ± 5.26 ^B^
6	8.02 ± 0.05 ^A^	450.46 ± 5.85 ^C^
8	8.11 ± 0.02 ^A^	23.11 ± 0.22 ^D^
10	8.07 ± 0.03 ^A^	ND ^E^

^1^ Data represent mean ± standard deviation determined by triplicate experiments. Mean values with different upper letters (A–E) in the same column are significantly different (*p* < 0.05) according to Fisher’s pairwise comparisons. ^2^ The correlation between NaCl concentration (X, %) and γ-PGA production (Y, μg/mL) was described by the quadratic equation Y = −17.14X^2^ + 77.20X + 534.83 (R = 0.98); the not-detected (ND) value at 10% NaCl was excluded from the regression.

## Data Availability

The datasets generated during and/or analyzed during the current study are available from the corresponding author upon reasonable request.

## Supplementary Materials

The following supporting information can be downloaded at https://www.mdpi.com/article/10.3390/antiox15080985/s1, Figure S1: Changes in (a) pH, (b) water activity, (c) total viable mesophilic bacterial counts, and (d) γ-PGA content during the fermentation of *Cheonggukjang* inoculated with γ-PGA-producing *Bacillus* strains under the conditions of 40 °C and 0% NaCl. ●: B group prepared without an inoculum (blank), ○: C1 group inoculated with *B. subtilis* KCTC 3135 (control 1), ■: C2 group inoculated with *B. velezensis* KCTC 13012 (control 2), □: S group inoculated with *B. velezensis* CH7P29. Error bars indicate standard deviations determined from triplicate experiments; Figure S2: Changes in (a) hardness, (b) adhesiveness, (c) springiness, (d) gumminess, (e) chewiness, and (f) cohesiveness during fermentation of *Cheonggukjang* inoculated with γ-PGA-producing Bacillus strains under optimal conditions, ●: B group prepared without an inoculum (blank), ○: C1 group inoculated with *B. subtilis* KCTC 3135 (control 1), ■: C2 group inoculated with *B. velezensis* KCTC 13012 (control 2), □: S group inoculated with *B. velezensis* CH7P29. Error bars indicate standard deviations determined from triplicate experiments; Table S1: Comparison of texture parameters between commercial product and optimally fermented *Cheonggukjang* samples inoculated with γ-PGA-producing *Bacillus* strains. C1 group inoculated with *B. subtilis* KCTC 3135 (control 1), C2 group inoculated with *B. velezensis* KCTC 13012 (control 2), S group inoculated with *B. velezensis* CH7P29. ^1^ Data represent mean ± standard deviation determined by triplicate experiments. Mean values with different upper letters (A–C) in the same column are significantly different (*p* < 0.05) according to Fisher’s pairwise comparisons.
