# Supplementary material for: Enhancement of Poly-γ-glutamic Acid Formation and Antioxidant Activity in Cheonggukjang Fermented with Bacillus velezensis CH7P29
Source: Antioxidants (Basel). 2026 Aug 9;15(8):985. doi: 10.3390/antiox15080985 (PMC13509646; doi:10.3390/antiox15080985)
Supplement: Supplementary file 1 [file antioxidants-15-00985-s001.zip › antioxidants-4426661-supplementary.pdf]

Figure S1 **Hong et al.**

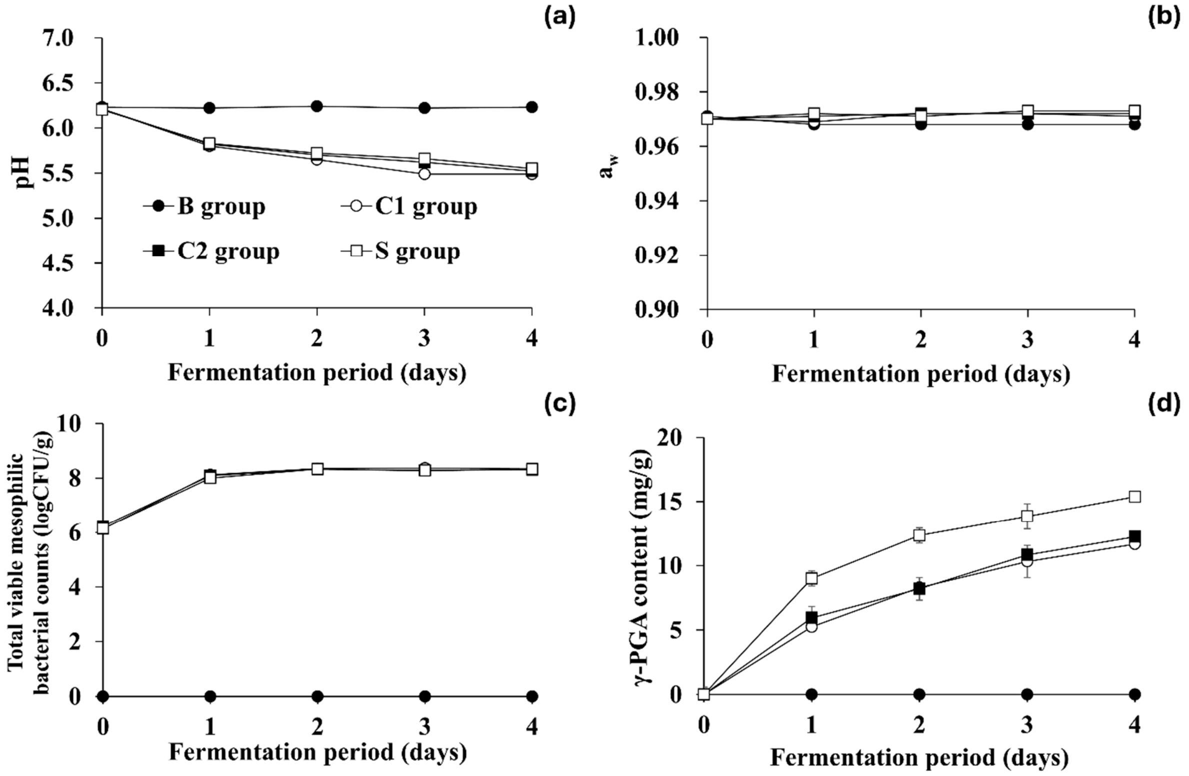

Figure S1. Changes in (a) pH, (b) water activity, (c) total viable mesophilic bacterial counts, and (d) γ-PGA content during fermentation of *Cheonggukjang* inoculated with γ-PGA-producing *Bacillus* strains under the conditions of 40 °C and 0% NaCl. ●: B group prepared without an inoculum (blank), ○: C1 group inoculated with *B. subtilis* KCTC 3135 (control 1), ■: C2 group inoculated with *B. velezensis* KCTC 13012 (control 2), □: S group inoculated with *B. velezensis* CH7P29. Error bars indicate standard deviations determined from triplicate experiments.

Figure S2 **Hong et al.**

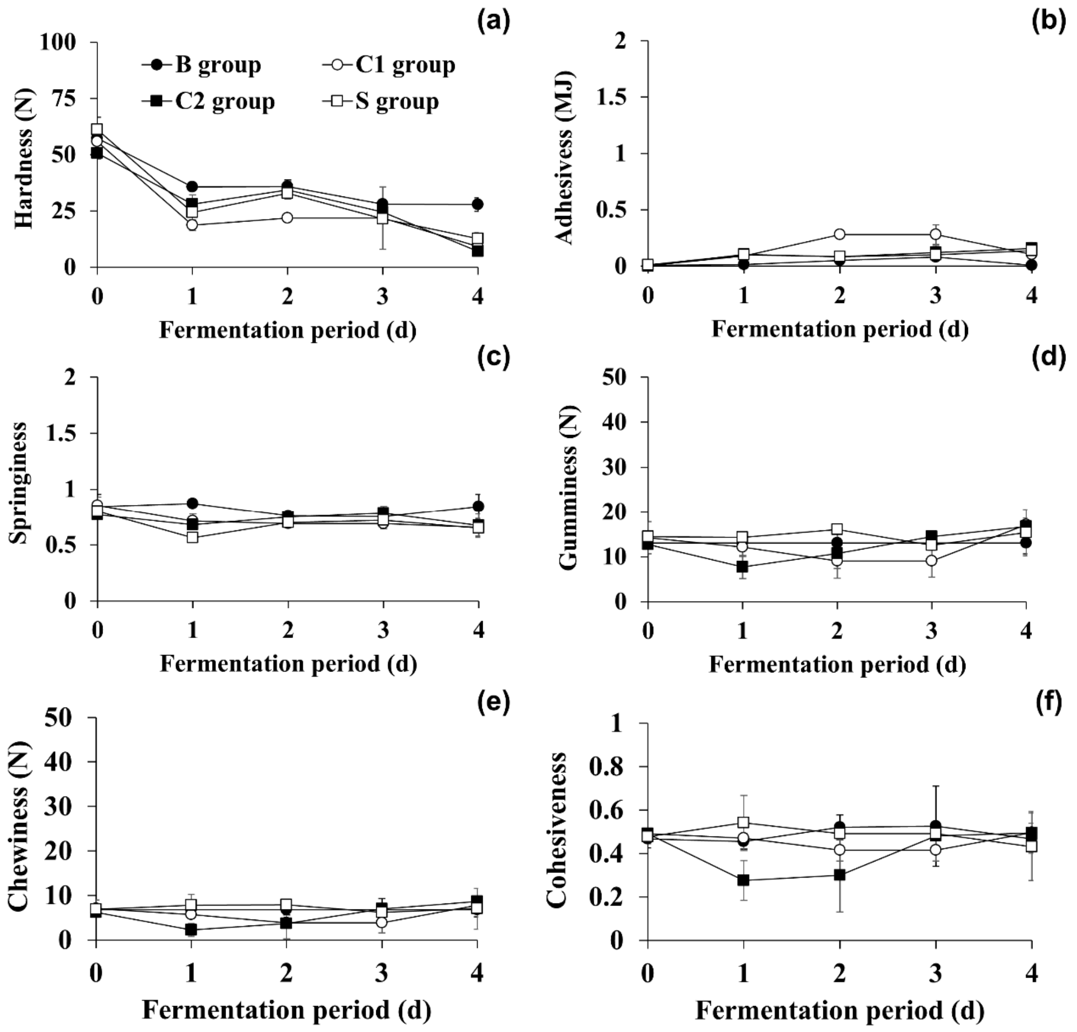

Figure S2. Changes in (a) hardness, (b) adhesiveness, (c) springiness, (d) gumminess, (e) chewiness, and (f) cohesiveness during fermentation of *Cheonggukjang* inoculated with γ-PGA-producing *Bacillus* strains under optimal conditions, ●: B group prepared without an inoculum (blank), ○: C1 group inoculated with *B. subtilis* KCTC 3135 (control 1), ■: C2 group inoculated with *B. velezensis* KCTC 13012 (control 2), □: S group inoculated with *B. velezensis* CH7P29. Error bars indicate standard deviations determined from triplicate experiments.

Table S1. Comparison of texture parameters between commercial product and optimally fermented *Cheonggukjang* samples inoculated with γ-PGA-producing *Bacillus* strains.

	Hardness (N)	Adhesiveness (MJ)	Springiness	Gumminess (N)	Chewiness (N)	Cohesiveness
Commercial product	5.71±0.95 $^{C, 1}$	0.14±0.01 ^{A}	0.61±0.07 ^{A}	16.59±0.96 ^{A}	7.01±0.53 ^{A}	0.43±0.03 ^{A}
C1 group	9.13±0.78 ^{B}	0.11±0.02 ^{A}	0.65±0.08 ^{A}	17.10±1.56 ^{A}	7.75±1.90 ^{A}	0.49±0.04 ^{A}
C2 group	6.98±1.05 ^{BC}	0.15±0.06 ^{A}	0.67±0.10 ^{A}	16.59±0.05 ^{A}	8.61±0.50 ^{A}	0.49±0.09 ^{A}
S group	12.77±2.65 ^{A}	0.14±0.04 ^{A}	0.65±0.07 ^{A}	16.59±5.14 ^{A}	7.01±4.60 ^{A}	0.43±0.15 ^{A}

C1 group inoculated with *B. subtilis* KCTC 3135 (control 1), C2 group inoculated with *B. velezensis* KCTC 13012 (control 2), S group inoculated with *B. velezensis* CH7P29. [1] Data represent mean ± standard deviation determined by triplicate experiments. Mean values with different upper letters (A–C) in the same column are significantly different ($p < 0.05$) according to Fisher's pairwise comparisons.
